# Bortezomib-inducible long non-coding RNA myocardial infarction associated transcript is an oncogene in multiple myeloma that suppresses miR-29b

**DOI:** 10.1038/s41419-019-1551-z

**Published:** 2019-04-09

**Authors:** Yunfeng Fu, Xiao Liu, Fangrong Zhang, Siyi Jiang, Jing Liu, Yanwei Luo

**Affiliations:** The Third Xiangya Hospital of Central South University, Changsha, 410013 China

## Abstract

Clinical outcomes of patients with multiple myeloma (MM) have almost doubled the overall survival over the last decade owing to the use of proteasome inhibitor such as bortezomib (BTZ). However, some patients with MM develop primary resistance to BTZ, whereas others develop resistance after treatment. In this study, we investigated relationships between BTZ resistance and dysfunction of long non-coding RNAs (lncRNAs) in patients with MM. Bone marrow samples were collected from patients with MM and healthy donors for lncRNA microarray and survival analyses. To investigate functions and underlying mechanisms of lncRNA-mediated BTZ resistance in MM, we performed CCK-8 assays, flow cytometry analyses, dual luciferase report gene assays, and RNA pulldown assays with samples from nude mice carrying tumor xenografts and in clinical samples. Differentially expressed lncRNA myocardial infarction associated transcripts (MIAT) were highly expressed in patients with MM compared with healthy controls, and were predictive of poor survival outcomes. Moreover, MIAT expression was significantly increased in BTZ-resistant patients with MM compared with newly diagnosed patients with MM, and was identified as a BTZ-inducible lncRNA. Specifically, BTZ upregulated MIAT expression through increased stat1 phosphorylation. Silencing of MIAT inhibited MM cell growth and sensitized MM cells to BTZ by negatively regulating miR-29b. Our data demonstrated the utility of MIAT as a tool for overcoming BTZ resistance in patients with MM.

## Introduction

Multiple myeloma (MM) accounts for approximately 10% of hematological malignancies^1^. Over the past decade, treatments with second-generation proteasome inhibitors, such as bortezomib (BTZ) and carfilzomib, have improved clinical outcomes for patients with MM and almost doubled overall survival^2^. Myeloma cells are sensitized to the inhibition of the 26S proteasome, resulting in the inhibition of NF-κB signaling^3^. However, owing to primary and acquired resistance to BTZ, most patients suffer relapse following treatment^4^. To address this challenge, genome-wide transcription studies of MM have identified multiple biomarkers that can be used for personalized treatments, including non-coding RNAs.

Long non-coding RNAs (lncRNAs) comprising more than 200 nucleotides^5^ participate in multiple biological processes involving epigenetic alterations. Moreover, dysfunctions of lncRNAs are associated with tumorigenesis and drug resistance in various cancers, including MM^6^. In particular, the lncRNA MALAT1 is overexpressed in MM and may be predictive of tumor progression^7,8^. Similarly, lncRNAs, such as CCAT1, H19, and NEAT1, have potential as biomarkers and treatment targets in patients with MM^9–11^, and NEAT1 knockdown reportedly improved dexamethasone sensitivity in patients with MM^10^. Moreover, Lu et al. recently showed that Linc00515 confers chemoresistance to melphalan-resistant myeloma cells by inhibiting miR-140-5p^12^. These studies warrant assessments of the roles of lncRNAs in BTZ resistance of MM.

In the current study, we identified differentially expressed lncRNAs in patients with MM. Among these, the lncRNA myocardial infarction associated transcript (MIAT) was highly expressed in patients with MM compared with healthy controls. Thus, we determined expression levels of MIAT in MM cells and the association of MIAT and prognosis of patients with MM were also investigated. In subsequent experiments, shRNA-mediated knockdown of MIAT sensitized MM cells to BTZ by regulating miR-29b. Our findings suggest that MIAT inhibition has potential as a therapeutic strategy for overcoming acquired BTZ resistance in patients with MM.

## Material and methods

### Tissue samples

Three MM and three healthy donor control bone marrow samples were collected for microarray analyses. Bone marrow tissues were obtained from an independent cohort of 143 patients with MM with clinical staging and survival information, 46 with newly diagnosed MM (NDMM), 34 with relapsed/refractory MM (RRMM), 35 with smoldering MM (SMM), and 28 with extramedullary myeloma (EMM) and 56 healthy donors. All tissue samples and corresponding clinical data were used in qPCR and survival analyses. Informed consent was obtained from each patient. This project was approved by the Ethics Committee of The Third Xiangya Hospital of Central South University.

### lncRNA microarray analysis

CD138+ plasma cells were collected from three patients with MM and three healthy donors (clinicopathological variables are shown in Table 1) and total RNA was extracted using RNeasy Mini Kits (Qiagen, GmBH, Hilden, Germany) according to the manufacturer’s instructions. After purifying total RNA, lncRNA expression profiles were analyzed by Aksomics Co. Ltd. (Shanghai, China) using human lncRNA Array V4.0 (8 × 60 K), which includes 35,923 lncRNAs and 24,881 coding genes. Raw data were then analyzed using GenePix 4000B and Gene-Spring software. Differentially expressed lncRNAs were identified as those with fold changes of ≥2 and *q* values of <0.05.

**Table 1:** Clinicopathological variables of patients with multiple myeloma and healthy donor used in microarray assay

Subjects	Age	Gender	Cytogenetic risk	Deletion 13q (%)	Deletion 17p (%)	t.(11·14) (%)	t.(14·16). (%)	DSS	ISS	IgH	IgL
MM patient 1	35	Male	High	Yes	No	No	No	I	I	IgG	κ
MM patient 2	48	Male	High	No	No	No	Yes	II	II	IgA	λ
MM patient 3	55	Male	High	No	Yes	No	No	III	III	IgG	λ
Healthy donor 1	32	Male	–	No	No	No	No	–	–	–	–
Healthy donor 2	45	Male	–	No	No	No	No	–	–	–	–
Healthy donor 3	58	Male	–	No	No	No	No	–	–	–	–

*DSS* Durie–Salmon staging, *ISS* international staging system

### Cell cultures and treatments

Myeloma cell lines (U266, KMS12, and KM3) were obtained from the National Infrastructure of Cell Line Resource (Beijing, China) and were cultured in RP1640 medium supplemented with 10% fetal bovine serum in an incubator containing 5% CO_2_ at 37 °C. A BTZ-resistant U266 cell line (U266/BTZ) was established in our lab by treating 1 × 10^5^ cells/ml with 1-nM BTZ. Media were changed once every 3 days and BTZ contents were maintained for 2 weeks, and were then doubled. After several iterations of dose doubling, cells were finally incubated in 30-nM BTZ.

Myeloma cell lines were exposed to BTZ at 0, 20, 40, and 80 nM for 24 h or at 40 nM for 0, 6, 12, or 24 h, or were exposed to the proteasome inhibitor MG132 at 0, 0.1, 0.5, or 1 μM for 24 h or at 0.5 μM for 0, 6, 12, or 24 h.

The inhibitors BTZ, MG132, SP600125 (c-Jun N-terminal kinase, JNK), U0126 (extracellular signal-regulated kinase, ERK), SB203580 (p38), Bay-11-7082 (NF-κB), and PF-04965842 (Janus kinase 1, Jak1) were purchased from Selleck (Shanghai, China). U266 cells were pretreated with these inhibitors for 2 h prior to further treatments and analyses.

### Cell infection and transfection

An optimal MIAT sequence was selected from three shRNAs and was recombined into a lentivirus vector to produce Lv-sh-MIAT. The target sequence was ACCCTGATCATTGCAAGGATCTCGTC. Lentiviral transduction particles containing shRNA for stat1 (TRC Number: TRCN000000426) were purchased from Merck (Shanghai, China). Cells (2 × 10^5^ cells per well) were seeded into culture wells and were cultured for 24 h. Upon reaching 80% confluence, cells were infected with lentivirus at a multiplicity of infection of 50 and were supplemented with 5-mg/ml polybrene (Sigma-Aldrich, St. Louis, MO, USA) for 48 h.

Plasmids expressing stat1 and empty pDONR223 plasmids were purchased from Youbio Co., Ltd. (cat no. G104301, Changsha, China). A nontargeting shRNA lentivirus expression vector was used as a negative control (Lv-sh-NC). Hsa-miR-29b inhibitors and mimics were purchased from RiboBio Co., Ltd. (Guangzhou, China) and were transfected into cells using Lipofectamine 3000 (Invitrogen) according to the manufacturer’s instructions.

### qRT-PCR

Total RNA was extracted using TRIzol reagent (Invitrogen). Reverse transcription was then performed using a maxima First Strand cDNA Synthesis kit (Thermo Fisher Scientific, Inc.) according to the manufacturer’s protocol. Quantitative polymerase chain reactions (qPCR) were then performed using a CFX96 Touch™ Deep Well Real-Time PCR Detection System (BioRad, Hercules, CA, USA). Mature miR-29b, miR-489, and miR-150 expression levels were determined using Hairpin-it^™^ miRNAs qPCR kits (Shanghai GenePharma Co., Ltd., Shanghai, China) according to the manufacturer’s protocol. Expression levels of the RNA U6 small nuclear 6 pseudogene were used as an internal control.

MIAT, Mcl-1, CDK-6, PSME4, and SP-1 expression levels were determined using UltraSYBR Mixture (CWBio, Wuhan, China) and β-actin expression was used as an internal control. Primers and their sequences are listed in Table 2. Thermocycling was performed with an initial denaturation at 95.0 °C for 3 min followed by 39 cycles of 95.0 °C for 10 s and 60 °C for 30 s. Relative gene expression was calculated using the comparative Ct method formula 2^−ΔΔCt^.

**Table 2:** The primer sequences used in qPCR

Gene	Sense (5′-3′)	Anti-sense (5′-3′)
MIAT	TCCCATTCCCGGAAGCTAGA	GAGGCATGAAATCACCCCCA
miR-29b	GCGTAGCACCATTTGAAATC	CAGTGCGTGTCGTGGAGT
miR-489	CTCAACTGGTGTCGTGGAGTC GGCAATTCAGTTGAGAGCTGC CGT	ACACTCCAGCTGGGGTGACATCACATA
miR-150	GAGGATCCCCGGGTACCGGTCTGGCAGGAACCCCCGCCCT	CACACATTCCACAGGCTAGTAAAAGCCGCA GCAGAGATG
Mcl-1	GGACATCAAAAACGAAGACG	GCAGCTTTCTTGGTTTATGG
CDK-6	CCGAGTAGTGCATCGCGATCTAA	CTTTGCCTAGTTCATCGATATC
PSME4	GGAGACCTTCTGCACTTCCAAGGATCTCAT	CCTCCCAAGTGTCTAAAGCCGCTTATACTG
SP-1	CCATACCCCTTAACCCCG	GAATTTTCACTAATGTTTCCCACC
U6	CTCGCTTCGGCAGCACA	AACGCTTCACGAATTTGCGT
β-actin	GCCCTATAAAACCCAGCGGC	TCGATGGGGTACTTCAGGGT

### Western blotting

Proteins were extracted using RIPA lysis buffer (Boster, Wuhan, China) and protein concentrations were determined using BCA protein assay kits (Thermo Scientific, Waltham, MA). Following separation on 10% sodium dodecyl sulfate-polyacrylamide gel electrophoresis (SDS-PAGE) gels, proteins were transferred onto polyvinylidene (PVDF) membranes and were immunoblotted with the following primary antibodies: phospho-Stat1 (Tyr701) antibody (Rabbit monoclonal, diluted at 1:1000, cat no. 9167, Cell Signaling Technology), phospho-Stat1 (Ser727) antibody (Rabbit monoclonal, diluted at 1:1000, cat no. 8826, Cell Signaling Technology), Stat1 antibody (Rabbit monoclonal, diluted at 1:1000, cat no. 14994, Cell Signaling Technology), p38 MAPK antibody (Rabbit monoclonal, diluted at 1:1000, cat no. 8690, Cell Signaling Technology), Bcl-2 antibody (Rabbit monoclonal, diluted at 1:1000, cat no. 4223, Cell Signaling Technology), Bak1 antibody (Rabbit monoclonal, diluted at 1:1000, cat no. 12105, Cell Signaling Technology), cleaved PARP antibody (Rabbit monoclonal, diluted at 1:1000, cat no. 5625, Cell Signaling Technology), total PARP antibody (Rabbit monoclonal, diluted at 1:1000, cat no. 9532, Cell Signaling Technology), Mcl-1 antibody (Rabbit monoclonal, diluted at 1:1000, cat no. 94296, Cell Signaling Technology), CDK-6 antibody (Rabbit monoclonal, diluted at 1:1000, cat no. 13331, Cell Signaling Technology), PSME4 antibody (Rabbit polyclonal, diluted at 1:1000, cat no. ABIN5533127, Abgent Biotech. (SuZhou) Co. Ltd., Suzhou, China), SP-1 antibody (Rabbit monoclonal, diluted at 1:1000, cat no. 9389, Cell Signaling Technology), and GAPDH antibody (Rabbit polyclonal, diluted at 1:1000, cat no. 5174, Cell Signaling Technology). Membranes were then incubated with appropriate secondary antibodies and protein bands were visualized using a BioRad imaging system (Hercules, CA).

### Luciferase reporter assays

The MIAT promoter was cloned into a pGL3-Basic vector using a Fast-Fusion™ Cloning Kit (FulenGen, Guangzhou, China). After treating with BTZ or transfecting with Lv-sh-Stat1, U266 cells were co-transfected with the internal control plasmid pRL-TK (Renilla luciferase reporter plasmid, Promega) and luciferase reporter constructs. Finally, luciferase activities were determined using a Dual Luciferase Reporter Assay Kit (Promega) according to the manufacturer’s instructions.

In further luciferase reporter assays, miRNAs (*n* = 200, Life Technologies) or negative control RNA were co-transfected with Renilla and firefly reporters with or without MIAT. Luciferase activities were then determined using Dual Luciferase Reporter Assay Kits (Promega) according to the manufacturer’s instructions.

To confirm the identities of target genes, the 3′ untranslated region (UTR) of miR-29b was amplified and subcloned immediately downstream of the luciferase gene sequence. Mutant MIAT (indicated by blue in Fig. 6f) was synthesized by Shanghai GenePharma Co., Ltd. Cells were then seeded and co-transfected with 100-ng aliquots of constructs with or without miR-29b mimics using Lipofectamine 3000 (Thermo Fisher Scientific, Inc.). Luciferase activity was detected as described above.

### CCK-8 assays

Cell viability was determined using CCK8 assays. After seeding at 5000 cells/well and culturing for 24 h, cells were transfected with the indicated miRNAs or lentivirus vectors for 48 h. Cells were then incubated for 24, 48, or 72 h, and 10-μl aliquots of CCK-8 reagents (Beyotime, Hangzhou, China) were added to each well. Optical density values were then measured using a microplate reader.

### Apoptosis analyses

After indicated treatments, cells were collected and washed twice in PBS and were then resuspended in 500-μl aliquots of binding buffer and incubated with 10-μl aliquots of Annexin V-FITC and 10-μl aliquots of propidium iodide (Propidium Iodide Annexin V apoptosis detection kit, Life Technologies, Grand Island, NY) for 15 min. Finally, the cells were analyzed using flow cytometry (BD Biosciences, San Jose, CA).

### Chromatin immunoprecipitation (ChIP) assays

Chromatin immunoprecipitation experiments were performed using EpiQuik Chromatin Immunoprecipitation Kits (EpiGentek, NY, USA). In these experiments, cells were lysed in RIPA lysis buffer. After centrifugation, supernatants were incubated with magnetic beads conjugated with antibodies (anti-Stat1, anti-Stat3, or anti-p65 antibody) overnight at 4 °C. Normal mouse IgG (Millipore) was used as a negative control. Finally, purified DNAs from precipitates were used for qPCR analyses.

### Pulldown assays with biotinylated MIAT and biotinylated miR-29b

Biotin labeling was performed using the Biotin RNA Labeling Mix (Roche Diagnostics, Indianapolis, IN) that was used for MIAT transcription. MIAT was then purified using RNeasy Mini Kits (Qiagen, Valencia, CA) according to the manufacturer’s protocol. Subsequently, the biotinylated MIAT probe was incubated with Nanobeads M-280 Streptavidin (Invitrogen, CA, USA) to generate probe-coated beads. U266 cell lysates were then incubated with these for 12 h and qPCR analyses were performed after eluting RNA complexes from the beads.

Biotinylated miR-29b, miR-29b-Mut, and negative control constructs were purchased from Ribobio (Guangzhou, China) and were used to transfect U266 cells for 48 h. After harvesting and lysing cells, lysates were incubated with Dynabeads M-280 Streptavidin (Invitrogen, CA, USA), and after elution from the beads, the RNA complexes were analyzed using qPCR.

### Tumor xenograft mice

All animal experiments were approved by the Ethics Committee for Animal Research of the Third Xiangya Hospital of Central South University. Mice were randomly divided into two groups and were injected with negative control transfected cells or sh-MIAT transfected cells by researchers who were blinded to the treatments. Transfected U266 cells (1 × 10^6^) were then subcutaneously injected into nude mice (*N* = 5) and tumor sizes were recorded periodically. Tumor volumes were calculated as 0.5 × *L* × *W*^2^, where *L* and *W* are long and short diameters of the tumor mass, respectively.

### Statistical analysis

Statistical analyses were performed using GraphPad Prism software (GraphPad Software Inc., La Jolla, CA). Significant differences were identified using Student’s *t*-test, one-way ANOVA with Bonferroni tests, Kaplan–Meier with log-rank tests, and Pearson’s correlation analyses, and were considered significant when *P* < 0.05.

## Results

### Differentially expressed lncRNAs in MM tissues

**Fig. 1:**
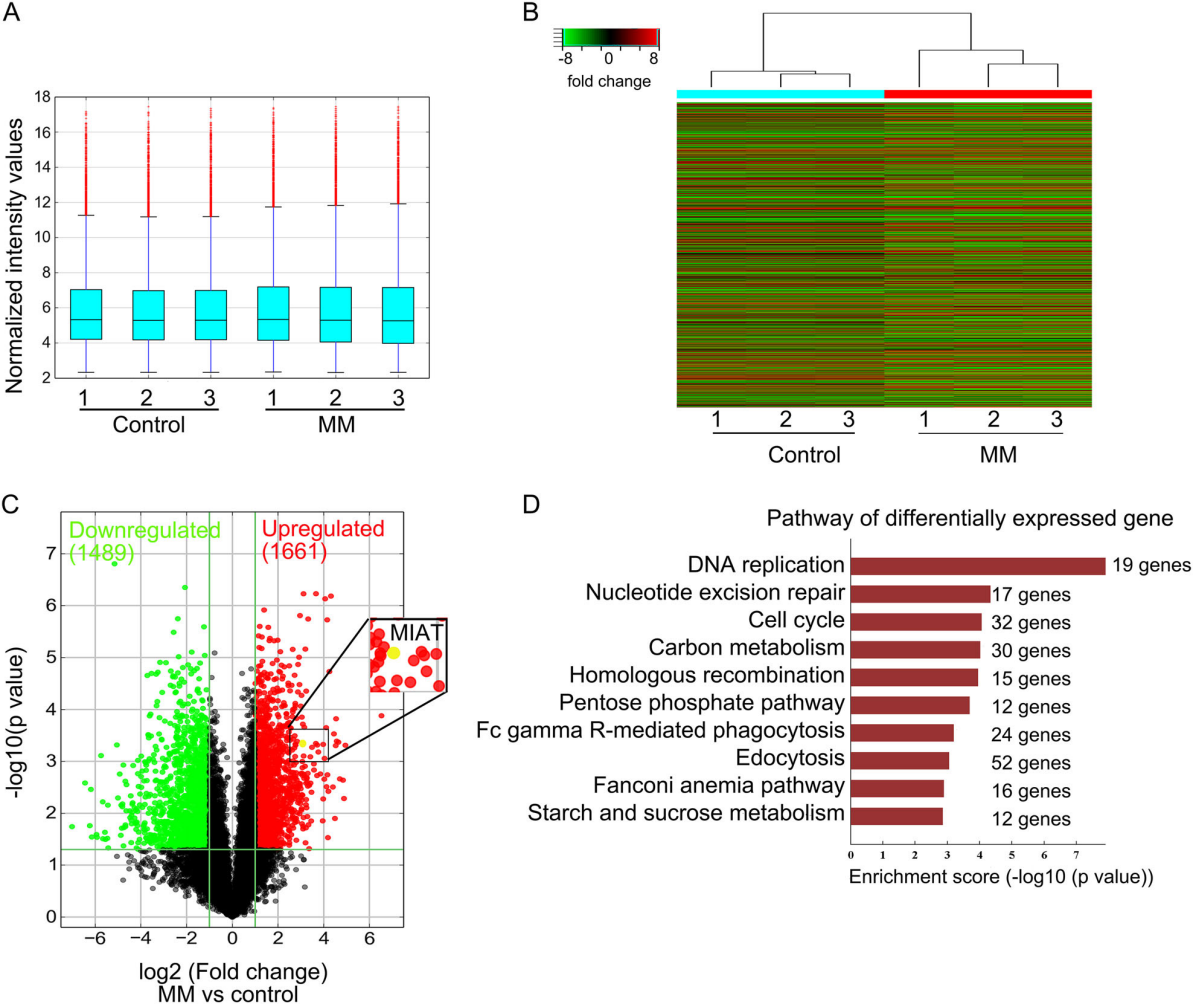
Differentially expressed lncRNAs in multiple myeloma (MM) plasma cells. Plasma cells from healthy donor controls and patients with MM were used to determine lncRNA profiles using a lncRNA expression microarray. **a** The box plot shows intensity values in control and MM groups. **b** The cluster heatmap shows lncRNAs with fold changes in expression of >2 from microarray data (*P* < 0.01). **c** A volcano plot is presented showing relationships between *q* values and magnitudes of differences in the expression values between subject groups. **d** Differentially expressed lncRNA-related pathways

To identify lncRNAs that are involved in MM development, we performed microarray analyses of bone marrow (BM) tissues from patients with MM and from healthy donors. Differentially expressed lncRNAs, including 1661 upregulated genes (red plots) and 1489 downregulated genes (green plots), are presented in volcano plots (Fig. 1a–c). lncRNA-MIAT was highly upregulated in BM tissues. Additionally, we analyzed the pathways involved in these lncRNAs. These differentially expressed lncRNAs were enriched in DNA replication, nucleotide excision repair, cell cycle, and carbon metabolism (Fig. 1d).

### MIAT expression is higher in MM plasma cells than in control cells

**Fig. 2:**
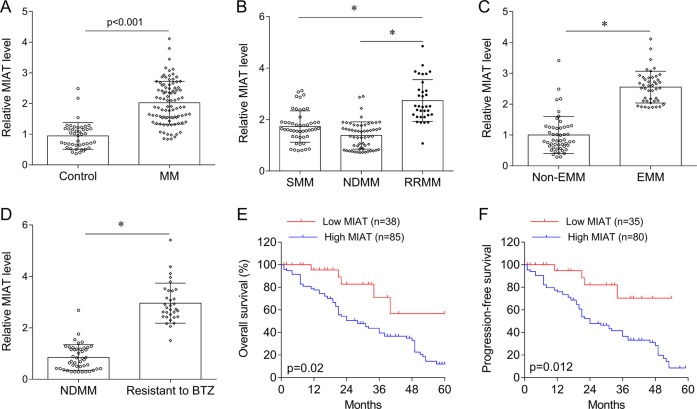
Myocardial infarction associated transcript (MIAT) expression in MM. **a** Quantitative reverse transcriptase-polymerase chain reaction (qRT-PCR) analyses of MIAT expression in control (*N* = 56) and MM tissues (*N* = 143). **b** MIAT in smoldering MM (SMM; *N* = 35), newly diagnosed MM (NDMM; *N* = 46), and relapsed/refractory (RRMM; *N* = 34) patients. **c** MIAT expression in extramedullary MM (EMM; *N* = 28) and non-EMM (*N* = 115) patients. **d** MIAT expression in NDMM (*N* = 46) and bortezomib (BTZ) resistant (*N* = 25) patients. **e** Overall survival analysis in patients with MM with low or high MIAT expression. **f** Progress-free survival analysis in patients with MM with low or high MIAT expression, *P < 0.05; NDMM newly diagnosed MM, RRMM relapsed/refractory MM, SMM smoldering multiple myeloma, EMM extramedullary myeloma

MIAT expression was significantly higher in MM plasma cells than in those from healthy controls (*P* < 0.001; Fig. 2a), and was also higher in patients with RRMM than in NDMM (*P* < 0.05) and SMM (*P* < 0.05) patients (Fig. 2b). MIAT expression in intramedullary plasma cells was higher in MM with EMM than in those without EMM (*P* < 0.05; Fig. 2c). MIAT expression was also significantly increased in patients with MM with BTZ resistance (*P* < 0.05, Fig. 2d). After dividing patients with MM into high and low MIAT expression groups using the median MIAT expression as a cut off, MIAT expression levels were significantly associated with cytogenetic risk (*P* = 0.001), DSS (*P* = 0.001), ISS (*P* = 0.001), IgH (*P* = 0.023), and IgL (*P* = 0.043), but were not associated with gender, age, or gene deletion and translocation (Table 3). Furthermore, in clinical assessments of the impact of MIAT expression in high and low MIAT expression groups from 123 patients with MM with overall survival (OS) data and 115 patients with MM with unavailable data, high MIAT expression was significantly associated with worse prognosis (Fig. 2e, f).

**Table 3:** Clinical association between MIAT levels and clinicopathological variables of patients with multiple myeloma

Variable	MIAT levels		*χ*^2^ test *P* value
	Low expression (*n* = 38)	High expression (*n* = 85)	
Age			0.836
<50	13	27	
≥50	25	58	
Gender			0.555
Male	24	48	
Female	14	37	
Cytogenetic risk (%)			0.001
High	14	59	
Standard	24	26	
Deletion 13q (%)			0.700
Yes	20	48	
No	18	37	
Deletion 17p (%)			0.845
Yes	22	51	
No	16	34	
t.(11·14) (%)			0.229
Yes	17	28	
No	21	57	
t.(14·16) (%)			0.890
Yes	13	28	
No	25	57	
DSS			0.001
I	15	12	
II	16	34	
III	7	39	
ISS			0.001
I	16	15	
II	17	33	
III	5	37	
IgH (%)			0.010
IgA	18	16	
IgD	0	4	
IgG	16	55	
IgM	1	6	
BJ	3	4	
IgL (%)			0.028
κ	26	40	
λ	12	45	

*DSS* Durie–Salmon staging, *ISS* international staging system

### MIAT is a BTZ-inducible lncRNA in MM cells

**Fig. 3:**
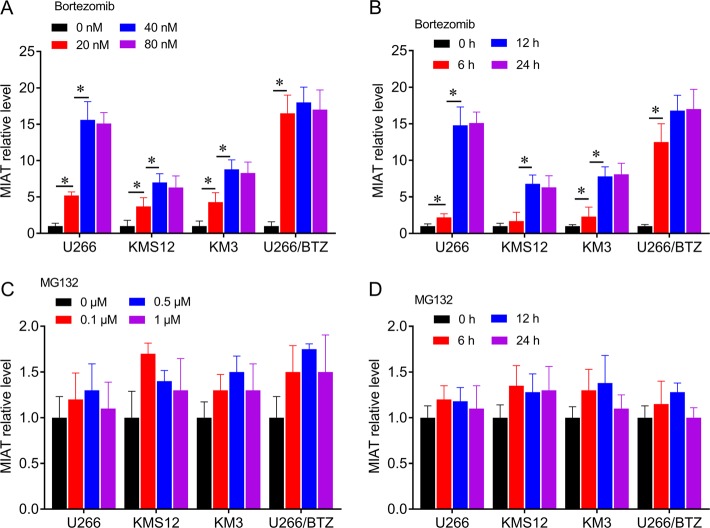
Changes in MIAT expression in response to BTZ and MG132 in myeloma cell lines. **a** Cells were treated for 24 h with BTZ at 0, 20, 40, and 80 nM. **b** Cells were treated with BTZ at 40 nM for 0, 6, 12, or 24 h. **c** Cells were treated for 24 h with MG132 at 0, 20, 40, and 80 nM. **d** Cells were treated with MG132 at 0.5 μM for 0, 6, 12, or 24 h, *P < 0.05

Because MIAT expression was greater in BTZ-resistant patients with MM than in NDMM patients, we confirmed responses of MIAT to BTZ using qRT-PCR analyses of MM cells. MIAT expression was also induced by BTZ in U266, KMS12, KM3, and U266/BTZ cells, and peaked at 12 h in the presence of 40-nM BTZ (Fig. 3a, b). In contrast, MG132 treatments did not lead to significant changes in MIAT expression in any of the cell lines (Fig. 3c, d).

### Upstream mediators of MIAT expression in MM cells

**Fig. 4:**
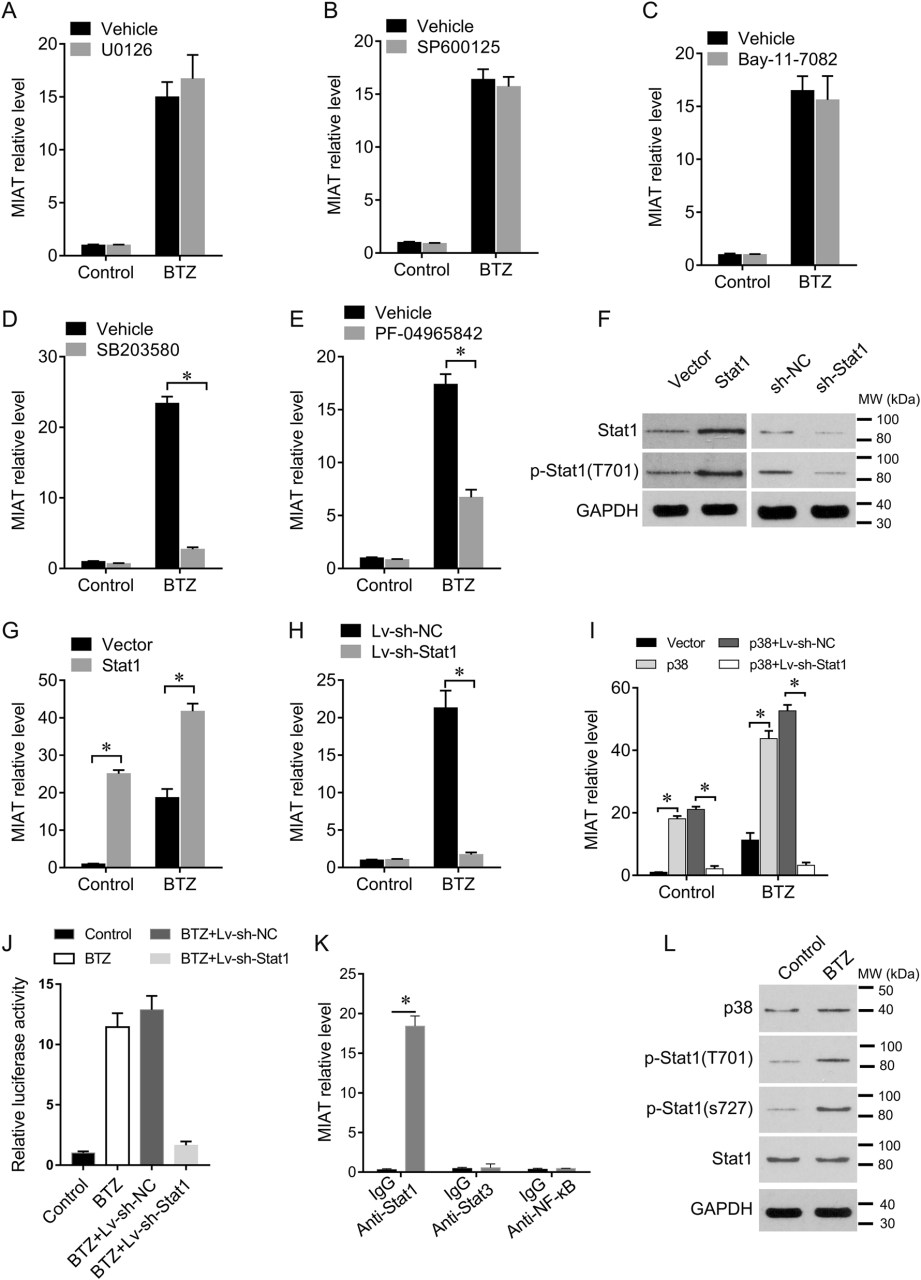
BTZ upregulates MIAT in MM cells via p38-Stat1 signaling. **a–e** U266 cells were pretreated with the selective pharmacological inhibitors U0126 (ERK, 50 μM), SP600125 (JNK, 50 μM), Bay-11-7082 (NF-κB, 10 μM), SB203580 (p38, 50 μM), or PF-04965842 (Jak1, 50 nM), and were then treated with BTZ at 40 nM for 12 h. MIAT expression levels were determined using qRT-PCR. **f** Western blotting analyses of Stat1 and phosphorylated Stat1 after overexpression or knockdown in U266 cells. **g** Effects of Stat1 overexpression on MIAT expression in U266 cells. **h** Effects of Stat1 knockdown on MIAT expression in U266 cells. **i** Effects of p38 overexpression on MIAT expression in U266 cells pretreated with sh-NC or sh-Stat1. **j** Luciferase reporter constructs containing the MIAT promoter were co-transfected into U266 cells with the internal control plasmid pRL-TK, and with sh-NC or sh-Stat1, and were then subjected to BTZ challenge (40 nM, 12 h). Relative luciferase activities are expressed as percentages of those in the control group. **k** Cell lysates from U266 cells were used for RIP with antibodies against stat1, stat3, or NF-κB. MIAT expression levels were detected using qRT-PCR. IgG was used as a negative control. **l** BTZ induced Stat1 phosphorylation; data are presented as means ± standard errors of the mean from three independent experiments; *P < 0.05; two-tailed pairwise comparisons were made with Student’s *t*-test

To distinguish signaling pathways through which BTZ inhibits MM cell growth, we performed experiments with various pathway inhibitors. Pretreatment with the ERK inhibitor SB203580 completely abrogated BTZ-mediated induction of MIAT, which was also partly reversed by the Jak1 inhibitor PF-04965842 (Fig. 4a–e).

Stat1 overexpression (Fig. 4f) also significantly enhanced BTZ-induced MIAT expression, whereas silencing of Stat1 suppressed BTZ-induced MIAT expression (Fig. 4g, h). Congruently, p38 overexpression increased MIAT induction by BTZ, and this effect was reversed by silencing Stat1 (Fig. 2i). BTZ treatments also significantly increased MIAT promoter activity, and Stat1 knockdown abrogated this effect (Fig. 4j). In addition, ChIP results showed that MIAT interacts with Stat1 but not with Stat3 and NF-κB (Fig. 3h). In accordance, BTZ treatments increased Stat1 phosphorylation at serine 727 and tyrosine 701 (Fig. 4l).

### MIAT knockdown inhibits MM cell growth

**Fig. 5:**
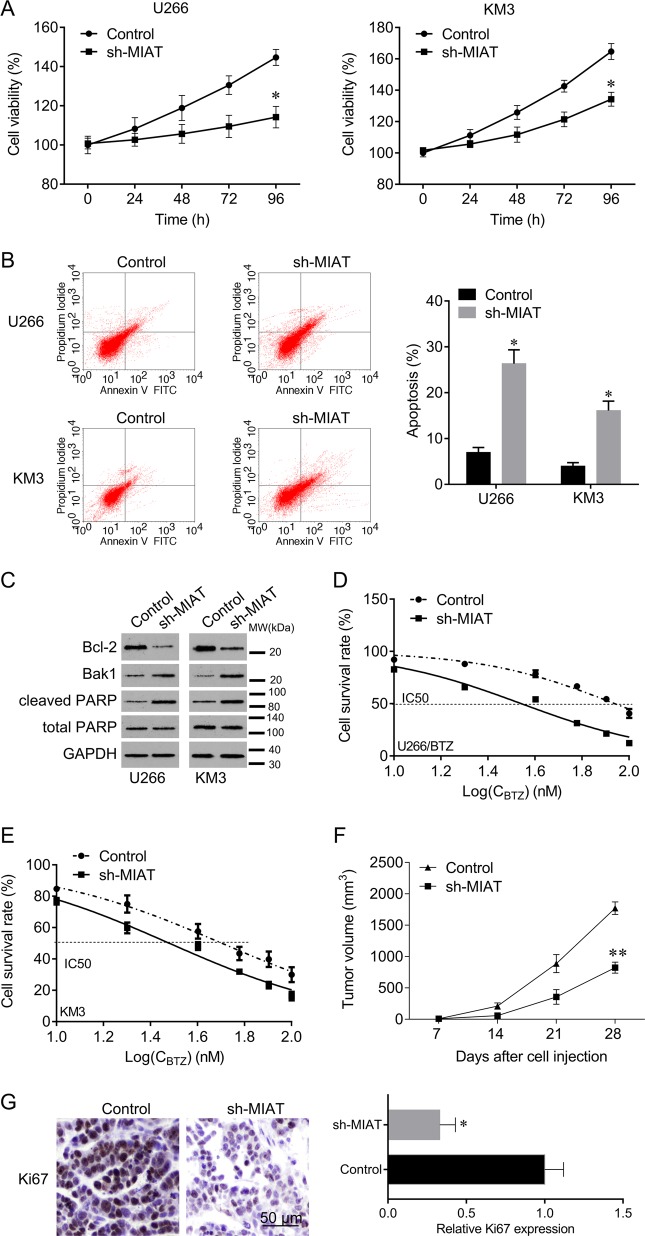
Knockdown of MIAT sensitizes MM cells to BTZ. **a** CCK-8 assays were used to determine cell viability after sh-MIAT lentivirus infection. **b** Flow cytometry was used to determine apoptosis after knockdown of MIAT. **c** Western blot analyses of apoptosis markers Bcl-2, Bak1, and cleaved PARP after knockdown of MIAT. **d** Changes of in half inhibitory concentrations (IC_50_) of BTZ in U266 cells after knockdown of MIAT were determined using CCK-8 assays. **e** Changes in IC_50_ of BTZ in KM3 cells after knockdown of MIAT. **f** U266 cells were infected with MIAT shRNA lentivirus and were then injected into nude mice. Cells transfected with empty lentivirus were used as a negative control. Mice were euthanized and tumors were collected on day 28 after injection. Tumor volumes were measured every week. **g** Ki67 expression in tumor sections was evaluated using immunohistochemistry (IHC); Bar, 50 μm; *P < 0.05, **P < 0.01

MIAT knockdown significantly inhibited cell proliferation and promoted apoptosis compared with that in the negative control (Fig. 5a, b). MIAT knockdown also reduced Bcl-2 expression and increased Bak1 and cleaved PARP expression (Fig. 5c). In addition, the half inhibitory concentrations (IC_50_) of BTZ in U266/BTZ and KM3 cells were significantly reduced by sh-MIAT lentivirus infection, compared with those in the negative control (Fig. 5d, e). Furthermore, MIAT knockdown significantly inhibited tumor growth in vivo and decreased the expression of the proliferation marker Ki67 (Fig. 5f, g). These data collectively indicate that knockdown of MIAT inhibits cell growth and mitigates BTZ resistance in MM cells.

### MIAT interacts with miR-29b and negatively regulates its expression

**Fig. 6:**
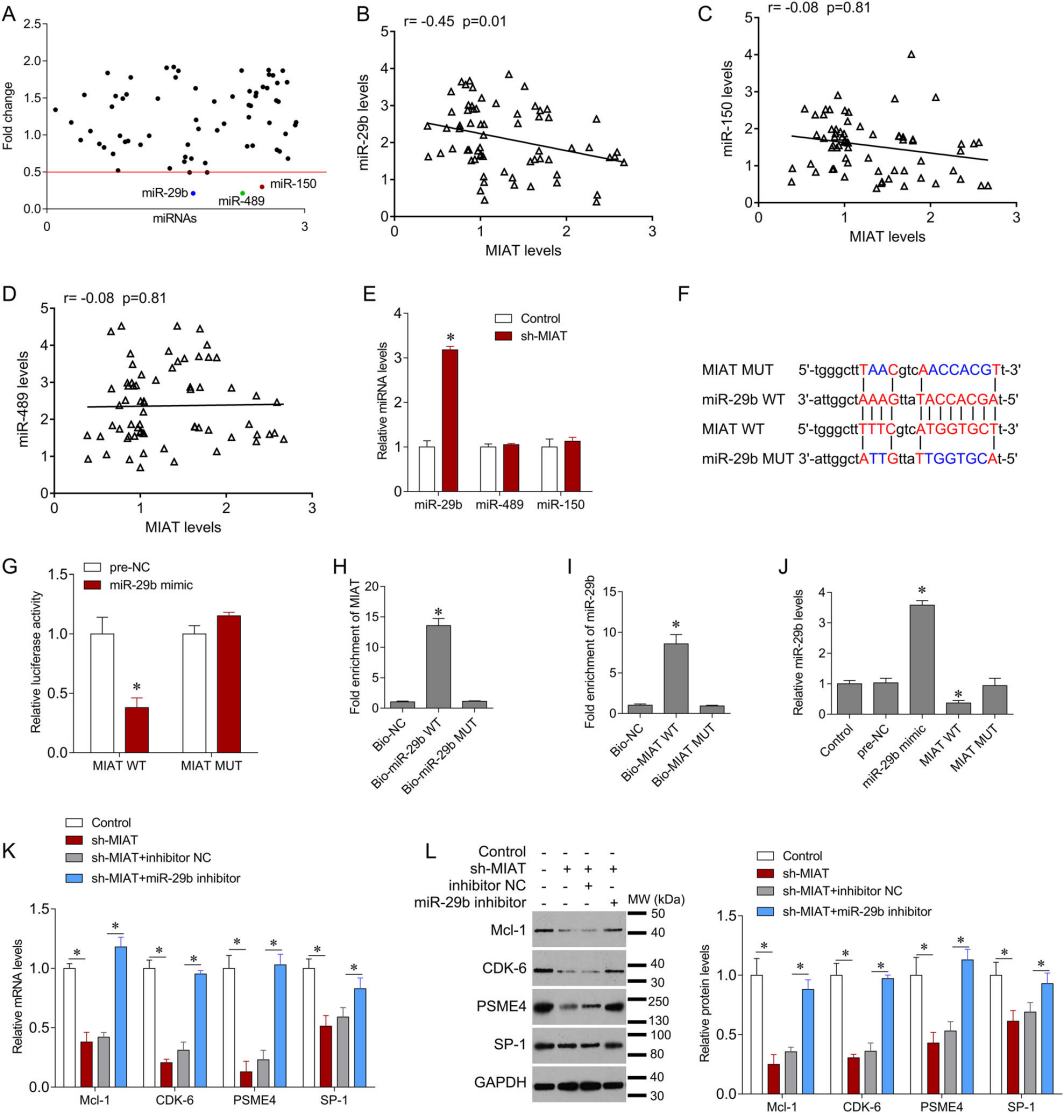
MIAT negatively regulates miR-29b. **a** Luciferase reporter gene assays of library miRNAs were performed to screen for MIAT targets. **b**–**d** Correlations between MIAT and miR-29b, miR-489, and miR-150 in patients with MM. **e** qRT-PCR analyses of miR-29b, miR-489, and miR-150 expression in U266 cells after infection with sh-MIAT lentivirus or empty control vector. **f** Sequence alignments of miR-29b with putative binding sites within wild type (WT; red) and mutant regions (blue) of MIAT. **g** Relative luciferase activities were inhibited in U266 cells that were co-transfected with the wild-type MIAT 3′UTR vector and a miR-29b mimic, but not with the mutant-type vector. Firefly luciferase activity was normalized to that of Renilla luciferase. **h** Levels of MIAT in samples that were pulled down using biotinylated miR-29b were measured using real-time PCR. **i** miR-29b expression levels in samples that were pulled down using biotinylated MIAT were measured using real-time PCR. **j** U266 cells were transfected with miR-29b mimic, or with wild type (WT) or mutant (MUT) MIAT for 48 h, and miR-29b expression was determined using real-time PCR. **k** Real-time PCR analysis of validated miR-29b targets after indicated treatments. **l** Western blotting analyses of validated miR-29b targets after indicated treatments; GAPDH was used a loading control; *P < 0.05

The miRNAs miR-29b, miR-489, and miR-150 had low luciferase activities and were screened as targets of MIAT (Fig. 6a). In correlation analyses of their expression levels and MIAT levels, only miR-29b was negatively correlated with MIAT (Fig. 6b–d). In addition, knockdown of MIAT significantly increased miR-29b expression but not that of miR-489 and miR-150 (Fig. 6e), further confirming the target relationship. Upregulation of miR-29b also decreased wild type (WT) MIAT-luciferase activity, but had no effect on mutant (MUT) MIAT luciferase activity (Fig. 6g). Accordingly, WT MIAT pulled miR-29b down, whereas MUT MIAT did not (Fig. 6h, i). Congruently, miR-29b expression was significantly decreased by WT MIAT but not by MUT MIAT in U266 cells (Fig. 6j). In further analyses of miR-29b target genes, we showed that knockdown of MIAT significantly reduces the expression of Mcl-1, CDK-6, PSME4, and SP-1 compared with controls, and an inhibitor of miR-29b reversed the reductions of Mcl-1, CDK-6, PSME4, and SP-1 that followed MIAT downregulation (Fig. 6k, l). These results suggest that MIAT functions as a miR-29b sponge and negatively regulates its expression in MM cells, leading to control over the expression of miR-29b target genes.

### MIAT silencing sensitizes MM cells to BTZ through miR-29b

**Fig. 7:**
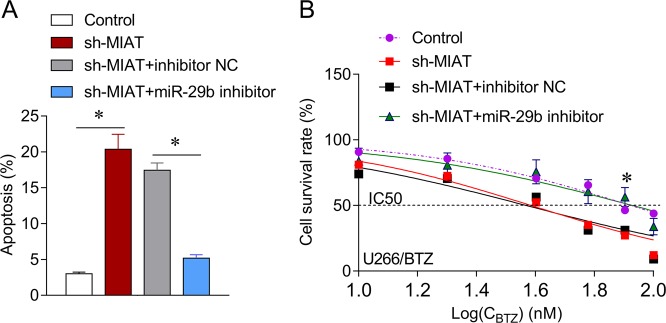
miR-29b inhibitor reverses the inhibitory effects of MIAT downregulation in U266 cells. **a** Apoptosis was evaluated using flow cytometry after treating U266 cells with miR-29b inhibitor and MIAT shRNA. **b** Changes in IC_50_ values for BTZ in U266 cells were determined using CCK-8 assays after treatments with miR-29b inhibitor and MIAT shRNA; *P < 0.05

Downregulated miR-29b repressed MIAT shRNA-mediated apoptosis (Fig. 7a). In addition, MIAT knockdown reduced the IC_50_ of BTZ in U266 cells, whereas the miR-29b inhibitor reversed this decrease (Fig. 7b). The results indicate that MIAT enhances BTZ resistance in MM cells by targeting miR-29b.

## Discussion

Our previous studies show that MIAT expression is upregulated in lung cancer tissues compared with adjacent tissues. In particular, increased MIAT expression was associated with advanced stages and shorter OS times of lung cancer patients^13^. The present data show that higher MIAT expression is predictive of reduced survival of patients with MM. MIAT expression was also significantly higher in BTZ-resistant patients with MM than in newly diagnosed patients with MM, and MIAT was identified as a BTZ-inducible lncRNA. We also show that BTZ upregulates MIAT expression by increasing the phosphorylation of stat1. Conversely, silencing of MIAT inhibited MM cell growth and sensitized MM cells to BTZ by negatively regulating miR-29b.

MIAT (located at 22q12.1) was originally identified as a myocardial infarction susceptibility locus^14^, and its expression was reportedly upregulated in association with ischemic stroke, myocardial infarction, non-small-cell lung cancer, and chronic lymphocytic leukemia^15,16^. Hence, MIAT may provide a therapeutic target for several cancers, including prostate and non-small-cell lung cancers^17,18^. In other studies, MIAT was identified as an oncogenic lncRNA that promoted colorectal and gastric cancers by targeting miR-132 and miR-141^19,20^. Our data show for the first time that MIAT is highly expressed in patients with MM and that BTZ treatment induces MIAT expression.

In previous investigations, the antitumor effects of BTZ were mediated by several pathways, including those involving NF-κB, ERK, and JNK^21–23^. Moreover, BTZ inhibited both canonical and noncanonical activation of NF-κB in MM cells. Yet, atypical NF-κB activation can promote BTZ resistance in MM cells^24^. Nonetheless, we found that co-treatments with BTZ and inhibitors of NF-κB, ERK, or JNK did not affect MIAT expression. But co-treatment with BTZ and a p38 inhibitor significantly suppressed MIAT expression, and the Jak1 inhibitor partially reversed BTZ-induced MIAT expression. Hence, BTZ resistance may result in constitutive activation of ERK, NF-кB, and stat1 pathways^25,26^. Similarly, p38 may promote drug resistance in patients with MM by regulating ERK, NF-кB, and stat1 pathways^27,28^. Constitutive nuclear localization and transcription of Stat1 activates downstream target genes. In a study by Shen et al., stat1 mediated LINC00174 expression in colorectal carcinomas^29^. Stat1 also interacts with lncRNA PLAC2 to inhibit cell proliferation and induce cell cycle arrest in gliomacells^30^. Herein, we concordantly demonstrated that stat1 interacts with the MIAT promoter and positively regulates MIAT expression.

Silencing of MIAT inhibited MM cell growth and sensitized MM cells to BTZ. According to the competitive endogenous RNA (ceRNA) hypothesis, mRNAs and lncRNAs communicate with each other by competing for binding sites on miRNAs. Congruently, Li et al. found that MIAT binds to miR-29-3p and upregulates the expression of HDAC4^31^. Our recent studies also showed that MIAT levels are negatively correlated with miR-29b in patients with MM, and MIAT inhibited miR-29b expression through a direct interaction, leading to increased expression of the miR-29b target genes Mcl-1, CDK-6, PSME4, and SP-1. The human miR-29b family includes miR-29a, miR-29b, and miR-29c, which are highly homologues in humans, mice, and rats^32^. The anti-MM activity of miR-29b has been studied extensively^33^, and its expression is reportedly decreased in primary malignant plasma cells and MM cell lines^34^. Moreover, enforced expression of miR-29b triggered in vitro anti-MM activity by targeting MCL1 and CDK6^35,36^. PSME4 encodes the proteasome activator PA200, and was also identified as a novel target of miR-29b in MM cells, and induction of miR-29b reversed PA200-reduced proteasome inhibition following treatments with BTZ^37^. In other studies, high SP1 activity was related to sustained survival and proliferation of MM^38^, and pharmacological or genetic inhibition of SP1 activated miR-29b transcription^39^. Notably, BTZ upregulates miR-29b^34^. In the present study, overexpressed miR-29b strongly increased growth inhibition and apoptosis in BTZ-treated MM cells, whereas miR-29b inhibition attenuated the anti-MM activity of BTZ^34^. In this study, we demonstrate a novel mechanism by which BTZ increases miR-29b expression. In particular, we show that BTZ induces MIAT through the p38–stat1 pathway. We further identified MIAT as a ceRNA for miR-29b and show that miR-29b inhibition attenuates sh-MIAT anti-MM activity. However, further investigations are required to clarify the mechanisms by which MIAT regulates miR-29b expression in MM, and these may be epigenetic, as shown in a recent study of MIAT and miR-34a expression^13^. A similar report shows that miR-29b levels are epigenetically regulated in hematologic malignancies, including MM^40^, further suggesting that epigenetic mechanisms contribute to the regulatory relationship between MIAT and miR-29b.

In summary, the data presented herein demonstrates that MIAT could be exploited as a tool for mitigating BTZ resistance in patients with MM.
